# PrEP uptake and delivery setting preferences among clients visiting six healthcare facilities in Eswatini

**DOI:** 10.1007/s10461-022-03646-0

**Published:** 2022-04-16

**Authors:** Maxime Inghels, Hae-Young Kim, Frank Tanser, Anita Hettema, Shannon A. McMahon, Catherine E. Oldenburg, Sindy Matse, Stefan Kohler, Pascal Geldsetzer, Till Bärnighausen

**Affiliations:** 1 Lincoln International Institute for Rural Health, University of Lincoln, Brayford Pool, LN6 7TS Lincoln, UK; 2 Centre Population et Développement, UMR 196 Paris Descartes – IRD), SageSud (ERL INSERM 1244), Institut de Recherche pour le Développement, Paris, France; 3 Africa Health Research Institute, KwaZulu-Natal, South Africa; 4 Department of Population Health, New York University Grossman School of Medicine, New York, NY USA; 5 KwaZulu-Natal Innovation and Sequencing Platform, KwaZulu‐Natal, South Africa; 6 School of Nursing and Public Health, University of KwaZulu-Natal, Durban, South Africa; 7 Centre for the AIDS Programme of Research in South Africa (CAPRISA), University of KwaZulu-Natal, Durban, South Africa; 8 Clinton Health Access Initiative, H100 Mbabane, Eswatini Swaziland; 9 Heidelberg Institute of Global Health (HIGH), Heidelberg University, 69120 Heidelberg, Germany; 10 Bloomberg School of Public Health, Johns Hopkins University, 21205 Baltimore, MD USA; 11 Department of Ophthalmology, University of California, San Francisco, 94115 San Francisco, CA USA; 12 Francis I. Proctor Foundation, University of California, San Francisco, 94122 San Francisco, CA USA; 13 Department of Epidemiology and Biostatistics, University of California, 94158 San Francisco, CA USA; 14 Eswatini Ministry of Health, Mbabane, Eswatini Swaziland; 15 Division of Primary Care and Population Health, Department of Medicine, Stanford University, Stanford, CA United States; 16 Institute of Social Medicine, Epidemiology and Health Economics, Charité?Universitätsmedizin Berlin, corporate member of Freie Universität Berlin and Humboldt-Universität zu Berlin, Berlin, Germany

**Keywords:** Pre-Exposure Prophylaxis, HIV, Consumer preference, Health services, Eswatini

## Abstract

Due to the high HIV incidence among the general population of Eswatini, pre-exposure prophylaxis (PrEP) for HIV-exposed individuals is recommended. However, little is known about PrEP uptake and preferences in PrEP delivery healthcare setting among the general population. We conducted a secondary analysis of a randomized trial that aimed to increase PrEP uptake. All clients eligible for PrEP in one of six public-sector healthcare facilities in Eswatini were included. PrEP uptake was stratified by initial reason for visit (e.g. outpatient). Preferences in PrEP delivery setting were collected among those clients who initiated PrEP. A total of 1782 clients had their HIV acquisition risk assessed. Of these, 72% (1277/1782) were considered at risk by healthcare providers and, among them, 40% (517/1277) initiated PrEP. Uptake was higher among clients visiting specifically to initiate PrEP (93%), followed by HIV testing visits (45.8%) and outpatient visits (40%). Among those who initiated PrEP, preferred delivery settings were outpatient services (31%), HIV testing services (26%), family planning (21%) and antenatal services (14%). Men or those at high risk of HIV acquisition were more likely to prefer HIV testing and outpatient services, while young women were more likely to visit and express a preference for antenatal and family planning services. Outpatient services and HIV testing services could be preferable choices for PrEP delivery integration, due to the high PrEP uptake and delivery setting preferences of the populations who use these services. Antenatal and family planning could also be considered with a view to targeting the youngest women.

## Resumen

Debido a la alta incidencia del VIH entre la población general de Eswatini, se recomienda la profilaxis previa a la exposición (PrEP) para las personas expuestas al VIH. Sin embargo, se sabe poco sobre la aceptación de la PrEP y las preferencias en el ámbito de la atención sanitaria de la PrEP entre la población general. Se realizó un análisis secundario de un ensayo clínico que pretendía aumentar la aceptación de la PrEP. Se incluyó a todos los clientes elegibles para la PrEP en uno de los seis centros sanitarios del sector público de Eswatini. La aceptación de la PrEP se estratificó según el motivo inicial de la visita (por ejemplo, paciente externo). Se recogieron las preferencias en el entorno de administración de la PrEP entre aquellos clientes que iniciaron la PrEP. Se evaluó el riesgo de adquisición del VIH de un total de 1.782 clientes (de 2.238 contactados, el 80%). De ellos, el 72% (1277/1782) fueron considerados de riesgo por los profesionales sanitarios y, entre ellos, el 40% (517/1277) iniciaron la PrEP. El consumo fue mayor entre los clientes que acudieron específicamente para iniciar la PrEP (93%), seguido de las visitas para realizar la prueba del VIH (45,8%) y las visitas ambulatorias (40%). Entre los que iniciaron la PrEP, los entornos de prestación preferidos fueron los servicios ambulatorios (31%), los servicios de pruebas del VIH (26%), la planificación familiar (21%) y los servicios prenatales (14%). Los hombres o las personas con alto riesgo de contraer el VIH tenían más probabilidades de preferir las pruebas del VIH y los servicios ambulatorios, mientras que las mujeres jóvenes tenían más probabilidades de acudir a los servicios prenatales y de planificación familiar y expresar su preferencia por ellos. Los servicios ambulatorios y los servicios de pruebas del VIH podrían ser opciones preferibles para la integración de la entrega de la PrEP, debido a la alta aceptación de la PrEP y a las preferencias del entorno de entrega de las poblaciones que utilizan estos servicios. Los servicios prenatales y de planificación familiar también podrían considerarse con vistas a dirigirse a las mujeres más jóvenes.

## Background

It is now well established that pre-exposure prophylaxis (PrEP) is effective in the prevention of HIV acquisition among both men and women [1–4]. Although PrEP was initially recommended among men who have sex with men (MSM) as an additional prevention option, the World Health Organization (WHO) now recommends that PrEP should be delivered among any populations with an annual HIV incidence of more than 3% [5]. Because this 3% threshold is mainly met by highly HIV-exposed sub populations, PrEP activities have focused mainly on specific groups such as MSM or sex workers who are disproportionately affected by HIV [6]. Yet, in some population contexts in Southern and Eastern Africa, the high incidence of HIV justifies extending PrEP delivery more widely within the general population [7]. In sub-Saharan Africa, there is evidence that general populations are aware of and interested in PrEP [8, 9]. In two clinical trials, the uptake of PrEP in the general population were between 27% and 33% among eligible people [10, 11]. Yet, PrEP uptake and adherence could be further improved among those identified as HIV-exposed [10, 11].

Operational research on PrEP delivery has been well documented among sub-populations (e.g. MSM, sex workers), but remains scarce in general population. Existing studies suggest that groups from the general population prefer receiving PrEP from general health services rather than from a specialized clinic [12–14]. In the United States, a survey of 370 Black women accessing medical care for sexual health or urgent care needs showed they preferred PrEP delivery through “a regular source of healthcare” as opposed to sexually transmitted infection (STI) or family planning clinics [12]. Another US study shows that women seeking abortion preferred to receive PrEP through their usual primary care provider rather than through an abortion or sexually transmitted infection clinic [13]. A qualitative study among youth in South Africa shows that youth prefer PrEP delivery via clinic or pharmacy rather than through a youth center or doctor’s office [14]. These results differ from those observed among sub-populations such as sex workers or MSM who seems to prefer more specialized settings such as family planning clinics, non-governmental organizations or specialist consultations [15–17].

There is also paucity of evidence on the best way to integrate PrEP into existing health services. The few papers on this subject primarily consider specific sub-populations (e.g. MSM, sex worker) [18–20]. The delivery preferences of PrEP users could help to prioritize services where PrEP should be integrated with a view to improving PrEP users’ uptake and adherence.

In this article, we aim to describe the preferred health settings for PrEP delivery among PrEP initiators among clients visiting in six health facilities in Eswatini.

## Methods

### Setting

This study took place in Eswatini, a landlocked country in Southern Africa that holds the highest HIV prevalence worldwide, estimated at 27% among those aged 15 and over [21]. Despite a significant fall over the past decade, annual HIV incidence is still high among adults, estimated at 1.0% in men and 1.7% in women with many sub-groups above 3.o% [21, 22]. This study was part of a stepped-wedge randomized trial, the purpose of which was to measure the impact of a facility–based PrEP promotion package (e.g. PrEP promotion video, T-shirt, booklet) designed to increase PrEP uptake in the Eswatini population [11]. This study took place at six public-sector healthcare facilities in the Hhohho Region in Northwestern Eswatini between February 2018 and January 2019. Four were located in relatively remote rural areas (Horo, Ndvwabangeni Nazarene, Ndzingeni Nazarene, and Ntfonjeni) while the other two healthcare facilities were located in peri-urban areas (Hhukwini and Siphocosini) near to the capital, Mbabane. All sites provided HIV services, including free antiretroviral treatment (ART) and PrEP as well as other health services. Detailed characteristics of the health facilities involved have been published elsewhere [11].

### Design

None of the healthcare facilities included in the study ever provided PrEP services before the study. Healthcare workers (HCW) from each study site were trained to offer PrEP services (general information on PrEP, assessment of risk of acquiring HIV, the PrEP initiation process and monitoring of PrEP clients at follow-up visits). In Eswatini’s primary healthcare setting, HCWs routinely provide brief health education sessions to clients waiting at the healthcare facility in the morning. During these sessions, HCWs were asked to encourage people reporting HIV-negative or who did not know their HIV status to undergo an HIV test. Those who tested negative were provided standard HIV prevention package (e.g. counselling on HIV risk reduction, condoms) and basic information about PrEP. Those expressing an interest in using PrEP after this session underwent a risk assessment of acquiring HIV. This risk assessment was based on six questions related to behavior in the past 6 months: (i) Have you had unprotected (condom-less) sex?, (ii) have you had sex with partners who are HIV positive or whose HIV status you did not know?, (iii) have you had a sexually transmitted infection?, (iv) have you been using postexposure prophylaxis?, (v) have you had sex under the influence of alcohol and/or drugs?, and (vi) have you experienced or do you expect any situations that you consider to be risky for acquiring HIV? The benefits and limitations of PrEP were then discussed with those answering “yes” to any of these questions or with anyone willing to start PrEP regardless of the answer to the risk assessment tool. A joint decision between the client and the HCW was made on whether to initiate PrEP.

Among those accepting to start PrEP, exclusion criteria were as follow: unable to provide written informed consent, younger than 16 years old, having a suspected acute HIV infection, or having contraindications to tenofovir disoproxil fumarate (TDF) or lamivudine (3TC) as specified in the 2018 Eswatini Integrated HIV Management guidelines [23]. In addition, clients who stated that they would be unable to attend a follow-up visit around 1 month after initiating PrEP were asked to defer PrEP initiation until a later date when they would be able to return for this one month follow-up visit. The latter exclusion criterion was based on the Eswatini Ministry of Health’s willingness to make essential that every PrEP client should be able to attend their scheduled follow-up visits. Pregnancy was not considered either as an exclusion criterion or a clinical recommendation for PrEP initiation.

Enrolled participants then underwent a confirmatory HIV test (unless the participant had already had an HIV test on the day of PrEP initiation) [23], a point-of-care rapid test for hepatitis B surface antigen (HBsAg), and a laboratory-based serum creatinine count. Those testing positive for HBsAg also underwent a liver function test prior to PrEP initiation. PrEP, consisting of a daily combined oral pill of 300 mg TDF and 300 mg 3TC, was prescribed at the same visit during which the HIV risk assessment was conducted. The WHO and PEPFAR consider 3TC clinically interchangeable with emtricitabine (FTC) for PrEP [24, 25]. Generic 3TC/TDF is less expensive than FTC/TDF and already available in supply chains for HIV drugs.

At the initial phase of the study (i.e. control), PrEP was promoted through the morning counselling session in waiting room and availability of advertising material on PrEP (posters, one-page pamphlets and palm cards). During the intervention phase, five additions to initial phase were made: (i) a PrEP promotion video played in the waiting room each healthcare facility, (ii) all healthcare workers were provided with a T-shirt to wear promoting PrEP, (iii) healthcare workers were asked to use a detailed flipchart to guide their counselling about PrEP with all clients who were at risk for acquiring HIV and expressed an interest in possibly using PrEP, (iv) clients at risk and interested in PrEP were given a booklet for information and (v) a self-risk assessment form was displayed in the waiting room for clients to fill out and hand to a healthcare worker. This self-risk assessment form asked the same six questions to ascertain risk for acquiring HIV as the form used by the healthcare workers.

Additional details on the trial methodology have been published elsewhere [11].

### Data collected

Sociodemographic characteristics (sex, age, educational level, relationship situation), partner’s HIV status, sexual behavior in the past 6 month (condomless sex, sexually transmitted infection—STI, sex under the influence of alcohol and/or drugs) and reason for visiting (i.e., the health service they aim to attend) were collected for every individual completing the risk assessment tool. In addition, people who started PrEP were ask about the preferred service delivery point for PrEP refill and follow-up (among the following service: outpatient, HIV testing, antenatal care—ANC, postnatal care—PNC, STI, family planning—FP, child and welfare care or other services).

### Statistical analysis

**Fig. 1:**
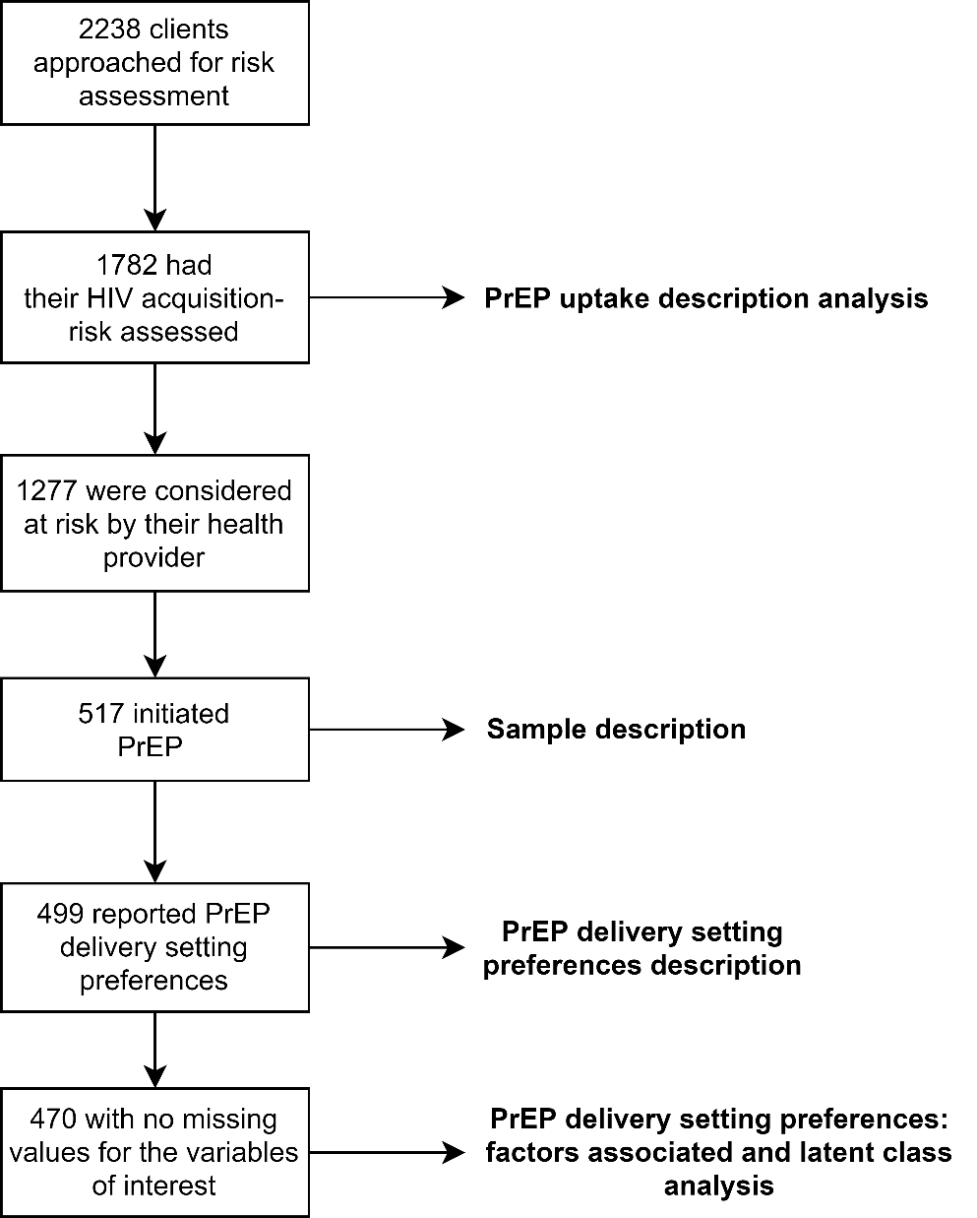
Sample selection process and statistical analysis conducted. PrEP: pre-exposure prophylaxis

Descriptive analysis was performed to measure PrEP uptake (number of people initiating PrEP divided by the total number of people seen at risk of HIV acquisition) and preferred service delivery point stratified by the reason of visit. Bivariate analysis was performed to measure the characteristics associated with PrEP setting preferences. P-values were computed for Pearson chi-squared test with Rao-Scott second-order correction. A cluster effect was added on the health facility. To investigate PrEP user profiles with PrEP delivery setting preferences, we conducted a latent class analysis. We included all the variables significantly associated to the 0.20 threshold from the previous bivariate analysis (i.e. associated factor with PrEP delivery setting preferences). Then, we conducted a backwards stepwise variable selection using regression model based on the minimisation of the Bayesian Information criterion (BIC) [26, 27]. The number of final classes was determined by both the BIC minimization and the classification quality (relative entropy). Classes founded in latent class analysis were compared by cluster-adjusted chi-square test of independence. All statistical analyses were computed using R version 3.6.3. using the package *survey*, for the cluster-adjusted analysis, *polCA* and *LCAvarsel* for the latent class analysis [28–30]. The Fig. 1 summarises the sample selection process for each analysis conducted (Fig. 1).

### Consent and ethical approvals

Every participant was informed about the study prior their participation. Written informed consent was collected from everyone accepting the offer to start PrEP. The study was approved by the Ministry of Health’s National Health Research Review Board (MH/599 C/IRB 0009688/NHRRB538/17) in Eswatini and the Chesapeake Institutional Review Board in the United States (Pro00021864). We received an exemption from ethical review by the Ethics Committee of the Faculty of Medicine at Heidelberg University in Germany.

## Results

### PrEP Uptake

**Fig. 2:**
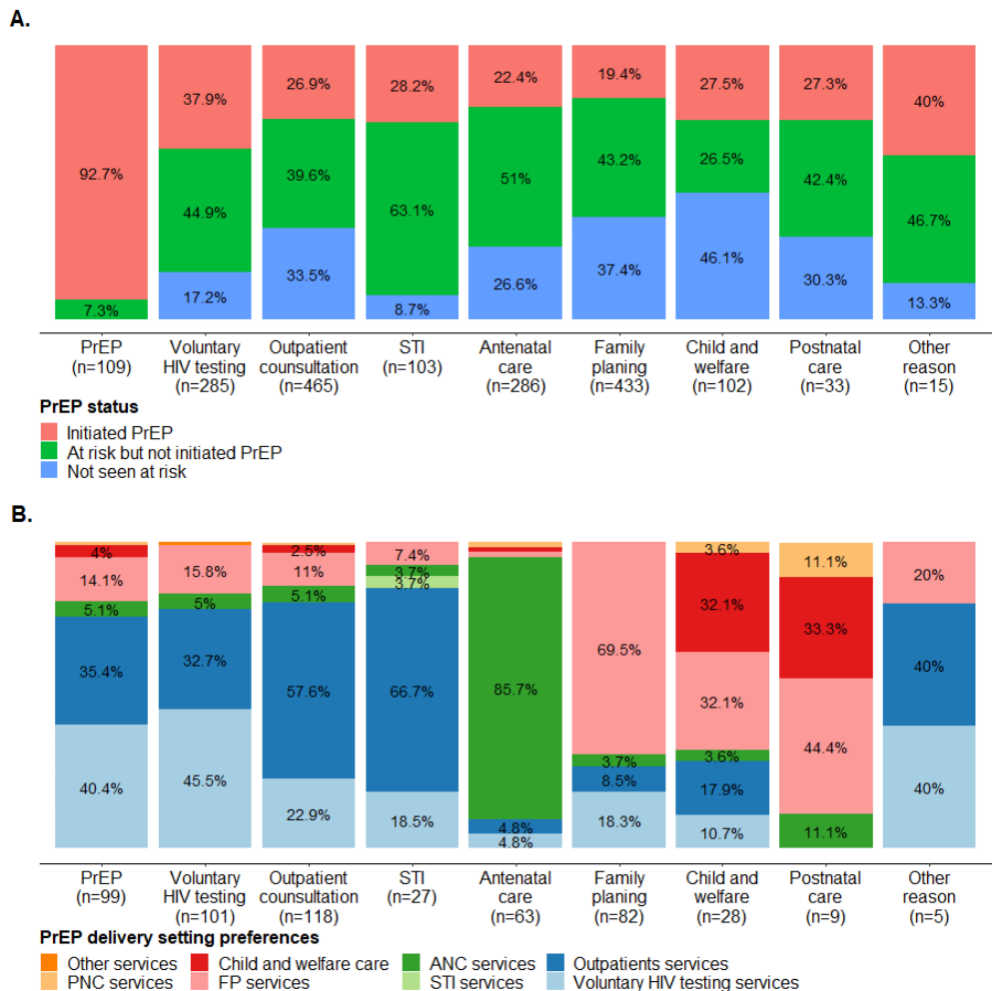
PrEP uptake (n = 1782) (A) and PrEP delivery setting preferences (n = 499)(B) by reason of visit. ANC: antenatal care, FP: family planning, PNC: postnatal care, PrEP: pre-exposure prophylaxis, STI: sexually transmitted infection. ANC: antenatal care, FP: family planning, PNC: postnatal care, PrEP: pre-exposure prophylaxis, STI: sexually transmitted infection. Note1: some clients reported more than one reason of visit, so the sum of the numbers is greater than the sample size. Note2: other reasons of visit included accompanying relatives or clients seeking post exposure prophylaxis for HIV

Overall, 2238 clients were approached for risk assessment in one of the six health centers. Among them, 1782 had their HIV acquisition-risk assessed. Among the 1277 who were considered at risk by their health provider, 517 initiated PrEP (Fig. 2, A).

Among the 1277 who were considered at risk, overall PrEP uptake was 40.5% (517/1277) with higher uptake among men (47.4%, 144/304) compared to women (38.4%, 372/972). However, in terms of absolute numbers, more women have initiated PrEP (372 vs. 144). PrEP uptake was higher for PrEP visit (92.7%, 101/109) and child and welfare care (50.9%, 28/55) followed by HIV testing (45.8%, 108/236) and outpatient visits (40.5%, 125/310). Lower PrEP uptake was found for individuals seeking antenatal care (30.5%, 64/210), STI (30.9%, 29/94) and family planning visits (31.0%, 84/271).

### PrEP delivery setting preferences

#### Sample characteristics

The table below describes the characteristics of the 517 who started the PrEP. This sample was mainly by women (72.1%) (Table 1). HIV acquisition exposure was high, 32.9% reported having an HIV-positive partner, 47.0% did not know the HIV status of their partner and 82.8% reported unprotected sex in the past 6 months. Only one client self-reported as being a sex worker and none as men having sex with other men.

**Table 1:** Description of people initiating PrEP (n = 517)

Characteristics	N (%)
**Age**	
16–25	167 (32.3%)
26–35	223 (43.1%)
36–45	92 (17.8%)
> 45	35 (6.8%)
**Sex**	
Male	144 (27.9%)
Female	373 (72.1%)
**Level of education**	
None	28 (5.4%)
Primary	135 (26.1%)
Secondary	311 (60.2%)
Tertiary	35 (6.8%)
Missing	8 (1.5%)
**Relationship situation**	
Single, no relationship	4 (0.8%)
One partner, not living together	240 (46.4%)
One partner, living together	205 (39.7%)
Multiple partners	68 (13.2%)
**Partner HIV status**	
Negative	101 (19.5%)
Positive	170 (32.9%)
Unknown	243 (47.0%)
Missing	3 (0.6%)
**Unprotected sex (< 6 months)**	
No	84 (16.2%)
Yes	428 (82.8%)
Missing	5 (1.0%)
**STI (< 6 months)**	
No	420 (81.2%)
Yes	91 (17.6%)
Missing	6 (1.2%)
**Had sex under the influence of alcohol and/or drugs (< 6 months)**	
No	454 (87.8%)
Yes	57 (11.0%)
Missing	6 (1.2%)
**Reason for visit**	
PrEP services	83 (16.1%)
Voluntary HIV testing services	91 (17.6%)
Outpatient services	108 (20.9%)
STI services	19 (3.7%)
ANC services	59 (11.4%)
FP services	77 (14.9%)
Child and welfare care	22 (4.3%)
Other reason	4 (0.8%)
multiple services	49 (9.5%)
Missing	5 (1.0%)
**Preferred service delivery point for PrEP**	
Voluntary HIV testing services	134 (25.9%)
Outpatient services	162 (31.3%)
STI services	1 (0.2%)
ANC services	70 (13.5%)
FP services	107 (20.7%)
Child and welfare care	20 (3.9%)
PNC services	4 (0.8%)
Other services	1 (0.2%)
Missing	18 (3.5%)
**Urban-rural classification of the healthcare facility visited**	
Peri-urban	164 (31.7%)
Rural	353 (68.3%)

ANC: antenatal care, FP: family planning, PNC: postnatal care, PrEP: pre-exposure prophylaxis, STI: sexually transmitted infection

Note 1: primary education in Eswatini begins at the age of six and is a 7-year program. secondary school (which includes high school) is a 5-year program

Note 2: other reasons of visit included accompanying relatives or clients seeking post exposure prophylaxis for HIV

Note 3: the client answering “Other services” for PrEP service delivery point mentioned a dedicated health service for PrEP

### PrEP delivery setting preferences and reason of visit

PrEP setting preferences varied by reason of visit (Fig. 2, B). The health service visited and the preferred service for PrEP delivery was mainly the same for individuals seeking antenatal care (85.7%), family planning (69.5%) and outpatient services (57.6%). Individuals seeking voluntary HIV testing reported PrEP preferences for both voluntary HIV testing (45.5%) and outpatient services (32.7%) for PrEP delivery while majority of people seeking STI services preferred outpatient services (66.7%).

### Factors associated with PrEP delivery setting preferences

Among the 517 who started PrEP, 18 did not mention any preferences and were excluded from the analysis. In addition, six individuals who mentioned preferring STI, PNC services or other services were excluded because of their insufficient number. Of the remaining, 4 were excluded because they did not report having any partners and 18 because of missing values, leading to 470 included in the final sample.

In the univariate analysis, sex, the relationship status, reporting sex under alcohol or drugs influence (< 6 months) and reason of visit were significantly associated with PrEP delivery setting preferences (Table 2). Outpatient services were preferred by men (54.8% vs. 23.8% among women, cluster-adjusted global χ^2^ = 5.3, p = 0.01), those reporting multiple sexual partners (62.7% vs. 27.7% among those reporting one partner, χ^2^ = 10.3, p = 0.01), STI (46.4% vs. 29.0%, χ^2^ = 2.1, p = 0.18) and sex under alcohol and/or drugs influence over the past 6 months (58.0% vs. 29.0%, χ^2^ = 4.6, p = 0.02). Antenatal (ANC) services, Family planning (FP) services and child and welfare care were reported almost exclusively by women.

**Table 2:** Factors associated with PrEP delivery setting preferences (n = 470)

	Preferred service delivery for PrEP							
	**Voluntary HIV testing services** **N = 129**			**Outpatients services** **N = 151**	**ANC services** **N = 67**	**FP services** **N = 103**	**Child and welfare care** **N = 20**	**χ** ^**2**^
**Sex**								5.3*
Male	44 (34.9%)			69 (54.8%)	4 (3.2%)	9 (7.1%)	0 (0.0%)	
Female	85 (24.7%)			82 (23.8%)	63 (18.3%)	94 (27.3%)	20 (5.8%)	
**Age**								2.6
16–25	30 (19.4%)			40 (25.8%)	36 (23.2%)	41 (26.5%)	8 (5.2%)	
26–35	59 (28.9%)			68 (33.3%)	26 (12.7%)	41 (20.1%)	10 (4.9%)	
36–45	26 (32.9%)			29 (36.7%)	5 (6.3%)	18 (22.8%)	1 (1.3%)	
> 45	14 (43.8%)			14 (43.8%)	0 (0.0%)	3 (9.4%)	1 (3.1%)	
**Level of education**								1.3
None	11 (40.7%)			11 (40.7%)	2 (7.4%)	3 (11.1%)	0 (0.0%)	
Primary	34 (28.3%)			36 (30.0%)	17 (14.2%)	27 (22.5%)	6 (5.0%)	
Secondary	69 (23.8%)			91 (31.4%)	46 (15.9%)	72 (24.8%)	12 (4.1%)	
Tertiary	15 (45.5%)			13 (39.4%)	2 (6.1%)	1 (3.0%)	2 (6.1%)	
**Relationship situation**								8.1**
One partner, not living together	49 (21.7%)			63 (27.9%)	42 (18.6%)	55 (24.3%)	17 (7.5%)	
One partner, living together	63 (34.1%)			51 (27.6%)	23 (12.4%)	46 (24.9%)	2 (1.1%)	
Multiple partners	17 (28.8%)			37 (62.7%)	2 (3.4%)	2 (3.4%)	1 (1.7%)	
**HIV status of the partner**								2.7
Negative	20 (20.8%)			25 (26.0%)	21 (21.9%)	26 (27.1%)	4 (4.2%)	
Positive	58 (37.2%)			53 (34.0%)	18 (11.5%)	23 (14.7%)	4 (2.6%)	
Unknown	51 (23.4%)			73 (33.5%)	28 (12.8%)	54 (24.8%)	12 (5.5%)	
**Unprotected sex (< 6 months)**								1.5
Yes	108 (27.6%)			123 (31.5%)	62 (15.9%)	83 (21.2%)	15 (3.8%)	
No	21 (26.6%)			28 (35.4%)	5 (6.3%)	20 (25.3%)	5 (6.3%)	
**STI (< 6 months)**								2.1
Yes	21 (25.0%)			39 (46.4%)	11 (13.1%)	12 (14.3%)	1 (1.2%)	
No	108 (28.0%)			112 (29.0%)	56 (14.5%)	91 (23.6%)	19 (4.9%)	
**Had sex under the influence of alcohol and/or drugs (< 6 months)**								4.6*
Yes	11 (22.0%)			29 (58.0%)	3 (6.0%)	5 (10.0%)	2 (4.0%)	
No	118 (28.1%)			122 (29.0%)	64 (15.2%)	98 (23.3%)	18 (4.3%)	
**Reason for visit**								11.0***
PrEP services		35 (43.8%)		29 (36.2%)	4 (5.0%)	8 (10.0%)	4 (5.0%)	
Voluntary HIV testing services		41 (49.4%)		26 (31.3%)	3 (3.6%)	13 (15.7%)	0 (0.0%)	
Outpatient services		22 (23.2%)		56 (58.9%)	3 (3.2%)	11 (11.6%)	3 (3.2%)	
STI services		3 (17.6%)		13 (76.5%)	0 (0.0%)	1 (5.9%)	0 (0.0%)	
ANC services		3 (5.5%)		3 (5.5%)	47 (85.5%)	1 (1.8%)	1 (1.8%)	
FP services		13 (18.1%)		6 (8.3%)	3 (4.2%)	50 (69.4%)	0 (0.0%)	
Child and welfare care		2 (9.5%)		4 (19.0%)	1 (4.8%)	5 (23.8%)	9 (42.9%)	
multiple or other services		10 (21.3%)		14 (29.8%)	6 (12.8%)	14 (29.8%)	3 (6.4%)	
**Urban-rural classification of the healthcare facility visited**								1.0
Peri-urban		42 (29.2%)		56 (38.9%)	10 (6.9%)	24 (16.7%)	12 (8.3%)	
Rural		87 (26.7%)		95 (29.1%)	57 (17.5%)	79 (24.2%)	8 (2.5%)	

*p-value < 0.05; **p-value < 0.01; ***p-value < 0.001

ANC: antenatal care, FP: family planning, PNC: postnatal care, PrEP: pre-exposure prophylaxis, STI: sexually transmitted infection

Note: cluster-adjusted global chi-square test of independence (i.e. both rows and columns comparison for each characteristics) were computed

### Latent class analysis of individual initiating PrEP

With the exception of the level of education and the urban-rural classification of the healthcare facility visited, all the variables from the Table 2 were included in our initial model. After the backwards selection, relationship status and the use of alcohol and/or drugs during sex variable were excluded. Thus, our final classification was based on the following variables: sex, age, HIV status of the partner, reporting an STI (< 6 months), reason of visit and PrEP delivery setting preferences. Three classes were obtained. The entropy measured in our model was 0.856.

Three PrEP user profile groups were found (Table 3). The main group, class 3 (57.0%), was mixed with men and women, older (33.9% were above 35 years), who reported higher risk of HIV acquisition compared to the two other groups (21.3% reporting multiple partners compared to 1.3% and 0.8% in the two other groups, 43.3% reporting an HIV positive partner compared to 20.5% and 19.4% in the class 1 and 2 respectively). Among people in class 3, reason of visit was mainly for PrEP, voluntary HIV testing and outpatient services and their preference for PrEP delivery was for either voluntary HIV testing services or outpatient services.

**Table 3:** Description of the three class profiles (n = 470)

	Class 1 N = 78	Class 2 N = 124	Class 3 N = 268	χ^2^
**Sex**				37.2***
Male	0 (0.0%)	0 (0.0%)	126 (47.0%)	
Female	78 (100.0%)	124 (100.0%)	142 (53.0%)	
**Age**				9.6**
16–25	44 (56.4%)	49 (39.5%)	62 (23.1%)	
26–35	31 (39.7%)	58 (46.8%)	115 (42.9%)	
36–45	3 (3.8%)	17 (13.7%)	59 (22.0%)	
> 45	0 (0.0%)	0 (0.0%)	32 (11.9%)	
**Level of education**				4.0*
None	2 (2.6%)	2 (1.6%)	23 (8.6%)	
Primary	21 (26.9%)	32 (25.8%)	67 (25.0%)	
Secondary	52 (66.7%)	87 (70.2%)	151 (56.3%)	
Tertiary	3 (3.8%)	3 (2.4%)	27 (10.1%)	
**Relationship situation**				13.9**
One partner, not living together	55 (70.5%)	69 (55.6%)	102 (38.1%)	
One partner, living together	22 (28.2%)	54 (43.5%)	109 (40.7%)	
Multiple partners	1 (1.3%)	1 (0.8%)	57 (21.3%)	
**HIV status of the partner**				8.6**
Negative	19 (24.4%)	36 (29.0%)	41 (15.3%)	
Positive	16 (20.5%)	24 (19.4%)	116 (43.3%)	
Unknown	43 (55.1%)	64 (51.6%)	111 (41.4%)	
**Unprotected sex (< 6 months)**				6.9*
Yes	73 (93.6%)	103 (83.1%)	215 (80.2%)	
No	5 (6.4%)	21 (16.9%)	53 (19.8%)	
**STI (< 6 months)**				6.0*
Yes	9 (11.5%)	11 (8.9%)	64 (23.9%)	
No	68 (88.5%)	113 (91.1%)	204 (76.1%)	
**had sex under the influence of alcohol and/or drugs (< 6 months)**				13.8**
Yes	3 (3.8%)	3 (2.4%)	44 (16.4%)	
No	75 (96.2%)	121 (97.6%)	224 (93.6%)	
**Reason for visit**				20.2***
PrEP services	1 (1.3%)	2 (1.6%)	77 (28.7%)	
Voluntary HIV testing services	0 (0.0%)	12 (19.7%)	71 (26.5%)	
Outpatient services	6 (7.7%)	9 (7.3%)	80 (29.9%)	
STI services	0 (0.0%)	0 (0.0%)	17 (6.3%)	
ANC services	53 (67.9%)	0 (0.0%)	2 (0.7%)	
FP services	0 (0.0%)	72 (58.1%)	0 (0.0%)	
Child and welfare care	10 (12.8%)	11 (8.9%)	0 (0.0%)	
multiple or other services	8 (10.3%)	18 (14.5%)	21 (7.8%)	
**Preferred service delivery point for PrEP**				3.1 × 10^15^***
Voluntary HIV testing services	3 (3.8%)	17 (13.7%)	109 (40.7%)	
Outpatient services	1 (1.3%)	14 (11.3%)	136 (50.7%)	
ANC services	57 (73.1%)	4 (3.2%)	6 (2.2%)	
FP services	1 (1.3%)	89 (71.8%)	13 (4.9%)	
Child and welfare care	16 (20.5%)	0 (0.0%)	4 (1.5%)	

*p-value < 0.05; **p-value < 0.01; ***p-value < 0.001

ANC: antenatal care, FP: family planning, PNC: postnatal care, PrEP: pre-exposure prophylaxis, STI: sexually transmitted infection

Note: cluster-adjusted global chi-square test of independence (i.e. both rows and columns comparison for each characteristics) were computed

The second group, class 2 (26.4%), was uniquely composed by women reporting lower HIV exposed situations compared to the other two groups (8.9% reported STI compared to 11.5% and 23.9% in the group 1 and 3 respectively), were mainly visiting for FP services (58.1%) and preferred FP services for PrEP delivery (71.8%).

The last group, class 1 (16.6%), was exclusively composed by women, who initially consult for ANC services (67.9%) and preferred ANC services for PrEP delivery (73.1%). This group was characterized by a young population (56.4% having below 26 years old), which does not live with their partner (70.5%) and report a higher rate of unprotected sex (93.6% vs. 83.1% and 80.2% in class 2 and 3 respectively).

## Discussion

Our study is one of the few to document PrEP uptake and delivery setting preferences among clients from the general population visiting a healthcare facility in Sub-Saharan Africa. We showed that both PrEP uptake and delivery preferences are linked to the reason of healthcare facility attendance. In addition, we have highlighted a variety of sociodemographic and behavioral profiles linked with each PrEP delivery setting preferences.

HIV testing and outpatient services had both high volumes and high rates of PrEP uptake compared to other health services. These two services were also more likely to be preferred for PrEP delivery by the PrEP user profile group found in the latent class analysis with the higher HIV-exposition (e.g. those reporting an HIV-infected partner, recent STI, sex under alcohol or substance). In addition, men and those who visited the health structure specifically for PrEP preferred HIV testing or outpatient services for PrEP delivery. For these reasons, both HIV testing and outpatient services should be considered for PrEP delivery integration to reach men and the most HIV-exposed populations.

PrEP uptake was one of the lowest in ANC services, but was similar to another survey conducted in Kenya [31]. PrEP initiation during pregnancy is of particular benefit to prevent vertical HIV transmission, as well as sexual acquisition during pregnancy and postpartum periods where women are more vulnerable to HIV [5, 32]. In our study, women seen in ANC services preferred ANC services for PrEP delivery. Thus, integration of PrEP delivery in ANC services could be relevant to reach pregnant women that may not be seen in other health services. Integrate PrEP into ANC services could also give the opportunity to increase PrEP uptake among men via their pregnant partner as this approach have been studied for other HIV prevention intervention such as couple HIV testing [33].

Family planning services recorded the lowest PrEP uptake. Yet, maintaining PrEP delivery in these services are relevant for several reasons. First, although most of them belonged to the risk group with the lowest HIV exposition in the latent class analysis, women attending these services reported a relatively high frequency of unprotected sex. Second, FP services contributed to a high number of PrEP initiation, 21% of the total number of PrEP initiators. Third, FP services are well adapted to offer PrEP as individuals willing to start a contraception method other than condom often indicate condomless sex behaviors and thus sexual exposure to HIV. Fourth, FP services were preferred for PrEP delivery among women initiating PrEP in these services. In addition, like ANC services, women initiating PrEP in FP services were more likely to be young (16–25 years) compared to the other services-a population who are targeted for HIV activities by national recommendations [21].

The association found between reason of visit and delivery setting preferences, especially for ANC, FP and outpatient services, could be due to the motivation to limit the number of health services visited. For example, women visiting ANC could be more likely to prefer the same service for PrEP refill as they are more likely to access this service for other health related need than other services. The only exception found was for people visiting for an STI who preferred outpatient and HIV testing services for PrEP delivery rather than STI services. People may prefer other services because they did not think to return regularly to STI service. If offering PrEP in STI consultations should be maintained because of the relatively high uptake, the follow-up and refill could be referred to other services such as outpatient or HIV-testing services.

While PrEP uptake was higher among men compared to women, the number of men on PrEP remains lower compared to women because they are less likely to visit a health center compared to women [34]. Alternative approaches to reach men out of healthcare structures are needed.

Qualitative interviews conducted in our study population showed that lower PrEP uptake among women could be due to their less decision-making autonomy or, for pregnant women, to their misunderstandings that PrEP could be harmful to their baby [34]. Interventions aiming to empower women and reduce misinformation about PrEP may help to improve uptake among pregnant women.

Our study has limitations. PrEP uptake and PrEP delivery setting preferences were collected from people visiting a health center which may have thus excluded or underestimated people less likely to visit a healthcare facility. However, our study included clients visiting the health center specifically for PrEP initiation, who might have been reached by outreach activity or by word of mouth. The PrEP delivery setting preferences of these people, mainly HIV testing and outpatient services, could inform on the preferences of people who do not regularly frequent health services. Another limitation is that we did not consider other potential places for PrEP refill and follow-up such as pharmacy or specific community health center [14]. A population-based survey among 2498 men and women in west Kenya showed that both men and women prefer getting PrEP in clinics (44%) followed by pharmacies (38%) [9]. As long as the staff receive specific training, pharmacies might be a convenient place for PrEP users for follow-up and could reduce workload on clinic staff.

Our study contributes to fulfill an existing literature gap on PrEP preferences among sub-groups of the general population that does not belong to the other well studied at-risk sub-populations (e.g. sex worker, men who have sex with men). While the few existing studies suggest that people from the general population tend to prefer general health services for PrEP delivery [12–14], our results have showed reported preference for either general (e.g. outpatient visit) or specialized health services (e.g. family planning) depending on client profiles. Thus, our results suggest that PrEP could be integrated to both general and specialized services to meet the various PrEP delivery setting preferences observed in clients attending health facilities.

## Conclusions

PrEP uptake, PrEP setting delivery preferences and client profiles vary according to the type of health service visited. Considering the high level of uptake and the high risk of HIV acquisition profile among PrEP initiators, outpatient and HIV-testing services could be considered for PrEP offer, delivery and follow-up. Antenatal care and family planning services could also be considered because they are visited by young women who expressed a preference for PrEP delivery within these settings.

Our survey mainly included client seeking non-PrEP related care, then further research on PrEP delivery preferences are needed especially among those not visiting health services.

## Data Availability

The de-identified dataset has already been made available for another paper on the Stanford Digital Repository at https://purl.stanford.edu/ws532pb8949.

## List of abbreviations

ANC: antenatal care.
FP: family planning.
HIV: human immunodeficiency virus.
PrEP: pre-exposure propylaxis.
STI: sexually transmitted infection.
